# Brazil Nut (*Bertholletia excelsa*) Beverage Processed by High-Pressure Homogenization: Changes in Main Components and Antioxidant Capacity during Cold Storage

**DOI:** 10.3390/molecules28124675

**Published:** 2023-06-09

**Authors:** Wilson Valerio Vasquez-Rojas, Diana Martín, Tiziana Fornari, M. Pilar Cano

**Affiliations:** 1 Department of Biotechnology and Microbiology of Foods, Institute of Food Science Research (CIAL) (CSIC-UAM), Nicolás Cabrera 9, 28049 Madrid, Spain; ing.valeriovasquez@gmail.com; 2 Department of Production and Characterization of Novel Foods, Institute of Food Science Research (CIAL) (CSIC-UAM), Nicolás Cabrera 9, 28049 Madrid, Spain; diana.martin@uam.es (D.M.); tiziana.fornari@uam.es (T.F.)

**Keywords:** Brazil nut, plant-based milk, ultra-high-pressure homogenization, phenolic compounds, minor lipids, minerals, antioxidant capacity

## Abstract

High-pressure homogenization (HPH) is an emerging technology for obtaining physical and microbial stability of plant-based milks, but there is little information on the effects of this technology on the phytochemical components of the processed plant food beverage and during its cold storage. The effect of three selected HPH treatments (180 MPa/25 °C, 150 MPa/55 °C, and 50 MPa/75 °C) and pasteurization (PAS) (63 °C, 20 min) on minor lipid constituents, total proteins, phenolic compounds, antioxidant capacity, and essential minerals of Brazil nut beverage (BNB) were studied. Additionally, the study of the possible changes in these constituents was carried out during cold storage at 5 °C for 21 days. The fatty acid profile (dominated by oleic acid and linoleic acid), free fatty acid content, protein, and essential minerals (notable source of Se and Cu) of the processed BNB remained almost stable to treatments (HPH and PAS). Specifically, reductions in squalene (22.7 to 26.4%) and γ-γ-tocopherol (28.4 to 36%) were observed in beverages processed via both non-thermal HPH and thermal PAS, but β-sitosterol remained unchanged. Total phenolics were reduced (24 to 30%) after both treatments, a factor that influenced the observed antioxidant capacity. The studied individual phenolics in BNB were gallic acid, catechin, epicatechin, catechin gallate, and ellagic acid, being the most abundant compounds. During cold storage (5 °C) up to 21 days, changes in the content of phytochemicals, minerals, and total proteins were not noticeable for any treated beverages, and no lipolysis processes were promoted. Therefore, after the application of HPH processing, Brazil nut beverage (BNB) maintained almost unaltered levels of bioactive compounds, essential minerals, total protein, and oxidative stability, remarkable characteristics for its potential development as a functional food.

## 1. Introduction

Brazil nut (*Bertholletia excelsa*) is considered a food with high nutritional and functional value. Worldwide, 69,658 tonnes of Brazil nuts are harvested each year. This amount is very modest when compared to the sales of most other tree nuts, such as almonds (1,171,268 tonnes), walnuts (795,843 tonnes), and cashews (706,979 tonnes). Just three countries are responsible for the total global production: Bolivia (78%), Peru (16%), and Brazil (6%) [1]. In retail, raw Brazil nuts are sold in packs, in dried fruit and nut mixtures, and in breakfast products. Food and personal care companies also process the nuts to use as an ingredient in various snacks, baked goods, confectioneries, and cosmetics. The consumption of Brazil nut has been related to the risk reduction of chronic disease, an effect attributed to high contents of oleic and linoleic acids, phenolic compounds, γ-tocopherol, and selenium, phytochemicals able of the modulation of the antioxidant and anti-inflammatory systems [2]. Epidemiological studies have observed improvements in glutathione peroxidase activity and selenium status after Brazil nut consumption, an effect linked to the formation of antioxidant selenoproteins, maintenance of homeostasis, regulation of immunity functions, reduction of oxidative stress, and prevention of DNA damage [3]. Despite the high lipid content in Brazil nut (~70%), the Brazil nut’s consumption did not increase dyslipidemia risk, and the blood lipid profile remained unchanged. There are even reports that found positive effects, including reduction in total cholesterol, increase in HDL-cholesterol, and reduction in LDL-cholesterol, suggesting a favorable effect on cardiovascular health [2].

Beverage production is one of the main derivatives of the Brazil nut and has an important growing opportunity in the market as an alternative to milk. Brazil nut beverage may be consumed for different reasons, whether due to intolerance to milk, vegetarian diet, awareness of animal suffering, or a search for healthy food [4]. Recently, work about Brazil nut beverage (BNB) has reported the outstanding levels of selenium, phenolic compounds (predominated by flavan-3-ols and hydroxybenzoic acids), and bioactive minor lipid components—as β-sitosterol, γ-tocopherol and squalene—indicating it to be a potentially healthy food [5]. Heat treatment is the traditional process for the reduction or elimination of microorganisms for vegetable beverages, but this technology can degrade bioactive compounds (vitamins, volatile compounds, phenolic compounds) and deteriorate nutrient compounds and sensory quality. Moreover, to stabilize the colloidal system of the beverage during its storage, the addition of stabilizing substances (emulsifiers) can be used [6]. High-pressure homogenization (HPH) is an emergent non-thermal technology able to inactivate microbes and produce fine particles improving the emulsion stability. The fluid is forced to pass through a gap, causing mechanical stress (shear, hydrodynamic, and cavitation effects) and a rapid rise in temperature, inducing cell disruption. Its effectiveness in reduction of microbial load and improvement of stability in the colloidal system in models and real matrices is well documented [7]. Studies about effects of HPH on bioactive compounds compared to heat treatment are reported mainly in fruit juices, showing mostly better extractability and preservative properties for bioactive compounds (such as ascorbic acid, carotenoids, flavonoids, and vitamins) depending on pressure, inlet temperature, and cycles applied [8]. Studies of bioactive compounds in HPH-treated plant-based milk are scarce. When soy milk was treated at 200 and 300 MPa in combination with different inlet temperatures (55, 65, and 75 °C), it was observed that the total phytosterol and isoflavone contents increased with increasing pressures and temperatures, but total γ-tocopherol tends to be susceptible to HPH process after [8]. A similar effect was observed in almond milk [9]. A previous study conducted by our research group verified that a high-pressure homogenization process (HPH) conducted at lower pressure than 200 MPa and at a temperature range of 25 to 75 °C was a treatment that made possible to achieve total inactivation of inoculated *E. coli* (~8 Log UFC/mL) in standardized Brazil nut beverage (with total lipid 2.9% w.f.) and a reduction in microbial load (mesophilic microorganisms) while also showing good physical stability and a high sensory property score [10].

In this sense, the main objectives of this study were to explore the possible effects of high-pressure homogenization (HPH) processing by combining pressure and inlet temperature in the processing conditions that exhibited the better microbial inactivation and to compare them to the pasteurization (PAS) process regarding the main nutrients and bioactive compounds of Brazil nut beverage after the treatments and during cold storage at 5 °C.

## 2. Results and Discussion

### 2.1. Effect of HPH Process and Pasteurization on Protein Content

For the first part of the study, the level of *E. coli* reduction (inoculated in the BNB) when HPH and thermal processes were applied was studied in order to validate the processes [10]. HPH treatments were applied using a high-pressure homogenizer with a maximum capacity of 200 MPa. Pressures of 50, 100, 150, and 180 MPa combined with inlet temperatures of 25, 55, and 75 °C were tested. In this previous study, the control sample was a BNB processed via thermal pasteurization conducted at 63 °C for 20 min, with a posterior cooling of the beverage to 5 °C. In the second part of this study, the best HPH conditions for *E. coli* reduction (≥5 log CFU/mL) were selected to conduct the study of the processed BNBs under cold storage for up to 21 days at 5 °C, evaluating the physical, microbiological, physicochemical, and sensory aspects. The three selected HPH process conditions for cold-pasteurizing BNB were T1: 50 MPa/75 °C, T2: 150 MPa/55 °C, and T3: 180 MPa/75 °C, as Vasquez-Rojas et al. reported [10].

HPH Brazil nut beverages showed no significant changes due to the processing. There were no significant differences in the protein content between untreated and treated beverages just after the processing (day 0) and during their cold storage at 5 °C for up to 21 days) (Table 1). The ranges of protein contents in the BNB were 1.05 to 1.14 g/100 mL, close to the previous reported data about the Brazil nut beverage processing, and 0.8 to 1.43 g/100 mL [4]. These values are above the average of the protein contents of many commercial plant-based milks (~0.7 mg/100 mL) [9]. The main effect of the HPH processing on the proteins of plant-based milk beverages is related to possible modifications of their functional properties, since when proteins are submitted to acidification, heating, or treatment with enzyme processes, their solubility, dispersion, and aggregation can be changed [6]. A recent previous study about the validation of high-pressure homogenization (HPH) process on the stability of the BNBs reported the formation of protein and lipid aggregates in HPH-treated BNB, verified by microscopy study and analysis of particles sized. This fact could be attributable to protein denaturation, but their smaller particle size allowed better physical stability under cold storage than pasteurized BNB [11]. Therefore, in this study, no significant quantitative changes in protein content were observed in HPH-treated beverages or in pasteurized beverages during cold storage time (5 °C), but the beverage could suffer modifications to its functional properties. Further research is now being conducted to study these potential changes in the functional proteins of the BNBs processed using HPH.

### 2.2. Effect of HPH Process and Pasteurization on Lipid Constituents

#### 2.2.1. Fatty Acid Profile

The fatty acids identified and quantified via GC-MS in BNBs are shown in Table 2. All processed BNBs showed no significant differences in their fatty acid profiles whether untreated or just processed with HPH and thermal processes (pasteurization) (day 0). The most abundant fatty acids present in the BNBs were unsaturated fatty acids (UFA), dominated by oleic acid (~40%) and linoleic acid (~34%). The amount of saturated fatty acids (SFA) was lower, with palmitic acid (~15%) and stearic acid (~10%). These values are similar to the obtained in previous studies about the validation of HPH process to stabilize the Brazil nut [5,10]. The predominant level of UFA (generally more than 70%) compared to SFA is common in vegetable milk alternatives, as is the case for soybean, rice, almond, and cashew beverages [12]. The predominance of oleic acid and linoleic acid in foods is considered favorable due to their potential health benefits, such as reducing LDL cholesterol, increasing HDL, and reducing cardiovascular risk [13]. The stability of fatty acids after processing was also reported in other published studies for soy milk and for almond milk treated with thermal processing and HPH [14,15]. In the present study, the processed BN beverages also did not suffer modifications to their fatty acid profiles or contents during cold storage (5 °C), showing a great stability of up to 21 days (Table 2). Fatty acid degradation is usually associated with oxidative and acidification processes, mainly caused by enzymes such as lipoxygenases or microbial spoilage [10,16], factors that were apparently controlled by HPH and pasteurization in BNBs.

#### 2.2.2. Free Fatty Acids

**Figure 1:**
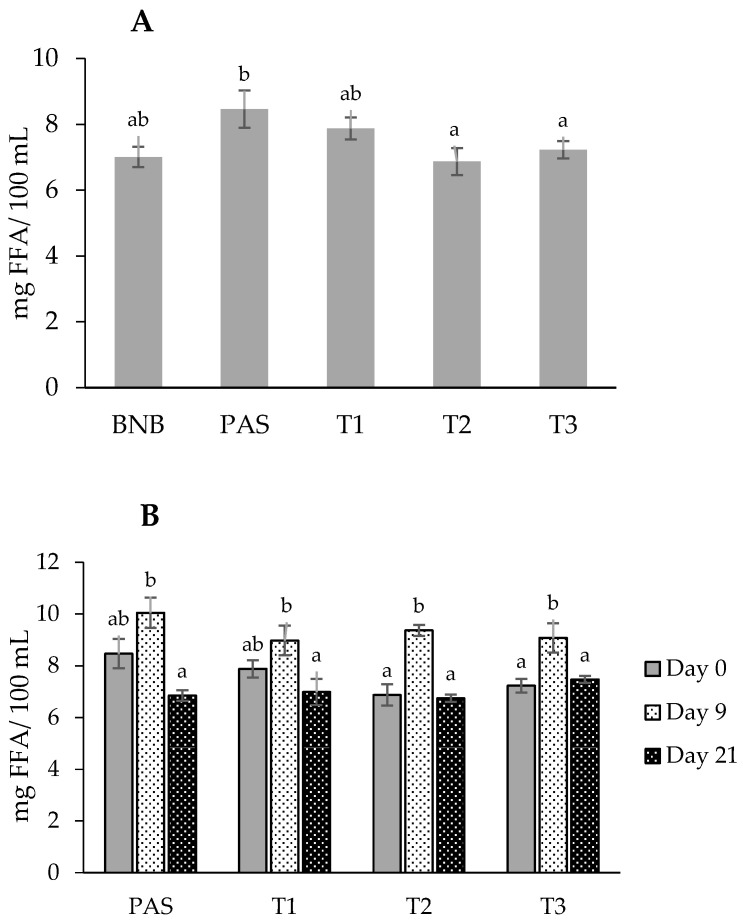
(**A**) Effect of processing (HPH and pasteurization) on FAA content (mg/100 mL) at day 0; and (**B**) evolution of FAA content during cold storage (5 °C). Data are expressed as mean ± standard deviation (*n* = 3). Different superscript lowercase letters indicate statistically significant differences (*p* ≤ 0.05) between conservation days within the same treatment.

In plant-based beverages the rancidity phenomenon is attributed to the lipolysis of triglycerides and an increase in free fatty acids (FFA). It is capable of producing bad taste and deterioration of the beverages. As observed in Figure 1A at day 0, HPH treatments did not modify the free fatty acid (FFA) content of BNB (6.87 to 7.88 mg/100 mL), but a significant increase was observed after the pasteurization process (8.47 mg/100 mL), Table S1, Supplementary Materials. These values are low if they were compared to the previous reported studies about Brazil nut beverages with FFA contents of 51.3 mg/100 mL (according to the GC-MS/MS method) [5] and between 200 to 300 mg/100 mL (according to the titratable acidity method, expressed in oleic acid) [16,17]. This noticeable difference contributed to the total lipid content, which is very high for a vegetable beverage. For this reason, in the present study, the BNB was partially defatted to obtain a better-balanced nutritional beverage [5]. In analogous vegetable beverages, such as soy milk and almond milk, the FFA contents also were high compared to the current work—i.e., 99 and 380 mg/100 mL (according to titratable acidity), respectively [16,18]. When expressed in a base oil, the FFA values of all samples were below 0.3 g/100 g oil, a value within the quality standards for virgin oil (≤2 g/100 g oil, expressed as oleic acid) established by the European community [19].

The effect of plant-based milk processing on FFA content was not previously reported, but it is known that the formation of hydroperoxides is closely associated to their degradation since the presence of FFA acts as a pro-oxidant, accelerating the rate of hydroperoxide decomposition. The studies conducted on soy milk, almond milk, and tiger nut milk reported that a slight or no increase of hydroperoxides, took place in the beverages after HPH and heat processing (pasteurization) [20,21,22]. Therefore, the processing via HPH or pasteurization of Brazil nut beverage (BNB) only slightly promoted an effect on lipolysis and lipid oxidation as was observed in the FAA data, Figure 1 and Table S1, Supplementary Material.

During cold storage, all BNBs showed a similar evolution of FFA content, Figure 1B. On day 9, there was a slight increase in FFA, probably due to lipolysis, and then a not-significant decrease until day 21—all values were within the narrow range from 6.8 to 9.4 mg/100 mL. The main factor associated with lipid hydrolysis is the action of lipases of plant or microbial origin. Seed enzymes are generally unstable and are destroyed by short pasteurization processes, but some—such as almond lipases—are stable over a wide temperature range (between 20 and 90 °C) [23]. Some bacterial lipases (such as those from pseudomonads) may also be heat-stable and thus may remain active after heat processing, which may contribute to lipolysis [24]. HPH can increase, stabilize, or decrease enzyme activity depending on the process conditions (pressure, temperature, and cycles) and the type of enzyme. Effects are linked to the reduction in particle size and modification of enzyme structures, altering their catalytic activity and the substrate available for enzymes [25]. For lipases, reports are scarce, but a study on milk observed that HPH at 200 MPa and inlet temperature 35 °C (outlet temperature around 71 °C) achieved total inactivation, equivalent to the thermal process (75 °C, 26 s) [26]. Considering that the pressure homogenization process can cause a temperature increase of about 2.5 °C/10 MPa [27], the combination of the HPH parameters (inlet temperature and pressure) applied to the Brazil nut beverage in the present study had an estimated valve temperature above 70 °C, a factor that could contribute to enzymatic inactivation and the stability of FFA content.

#### 2.2.3. Phytosterols, Tocopherols, and Squalene

These phytochemicals are the main bioactive compounds present in the lipid fraction of Brazil nut [5,28]. Therefore, in the present study, they were identified and quantified using GC-MS in HPH-processed Brazil nut beverages. The identification of the β-sitosterol and γ-γ-tocopherol was fast due to their abundance, as they are considered the main isomers in Brazil nut [29]. The abundance order of the bioactive lipids in Brazil nut beverage (BNB) was squalene (70.3 mg/L), followed by β-sitosterol (35.6 mg/L) and γ-γ-tocopherol (10.4 mg/L), Table 3. Compared to other plant-based beverages, β-sitosterol content in BNB was higher than in other commercial vegetable beverages (<30 mg/L) [30]. The γ-tocopherol level of BNB was higher than those of soy milk (3.5 mg/L) [7] and milk-fruit beverages (<5 mg/L) [31] but less than that of almond milk (50 mg/L, total tocopherol) [32]. In squalene, there are no previous reports, but considering the high squalene level of Brazil nut compared to most plant sources used for vegetable beverages [32], it is reasonable to say that BNB had a higher squalene level than other analogous beverages. Both processes, HPH and pasteurization, reduced the content of squalene (between 22.7 to 26.4%) and γ-γ-tocopherol (between 28.4 to 36%), possibly due to their thermolability, Table 3.

In general, the recommended optimal extraction temperature for the analysis of these compounds is below 60 °C, and at higher temperatures, they tend to undergo degradation [33,34]. On the other hand, as expected, the level of β-sitosterol showed no modification by HPH or pasteurization processes due to thermal stability up to around 140 °C, as reported for the extraction of phytosterols from oilseeds after roasting. A similar effect was also observed in HPH-treated almond milk and soybean milk at 200 and 300 MPa and Ti at 55 to 75 °C—high pressure/temperature intensity caused a significant reduction in γ-tocopherol and the stability (or relative increase) of phytosterol [8,33].

Table 3 also shows the evolution of these bioactive compounds drying after cold storage at 5 °C. In general, there was no significant effect of the process on the levels of squalene (between 45.8 to 57.5 mg/L), γ-γ-tocopherol (between 6.4 to 7.9 mg/L), or β-sitosterol (between 28.6 to 35.4 mg/L) in any treated BNB up to 21 days. Previous studies in other beverages (skim milk and fruit juice) also reported the stability of phytosterols up to a time of 6 months at different storage temperatures (4 to 37 °C) [35]. In addition, plant-based milks are considered a good matrix for preventing the possible formation of phytosterol oxidation products due to the presence of natural antioxidants [36], a factor that may have influenced the observed stability of β-sitosterol in Brazil nut beverages. There are no reports about the stability of γ-tocopherol and squalene of the vegetable beverages during cold storage, but it is known that factors such as fatty acid profile, storage temperature, exposure to light, and other oxidizing agents may influence them. In addition, γ-γ-tocopherol could be considered an effective lipid-soluble antioxidant that protects mono- and polyunsaturated fatty acids from oxidation [37], so the remarkable content of γ-γ-tocopherol present in Brazil nut beverages compared to other vegetable beverages [30] could explain their stability.

### 2.3. Effect of HPH Process and Pasteurization on the Phenolic Compounds

**Figure 2:**
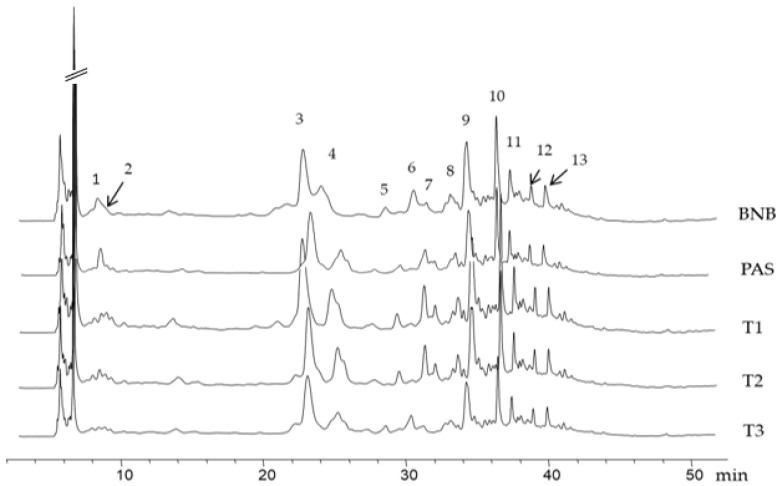
HPLC C18 chromatogram of phenolic compounds from Brazil nut beverage detected at 280 nm: (1) gallic acid, (2) gallic acid derivative, (3) catechin, (4) catechin derivative, (5) 4-hydroxybenzoic acid, (6) vanillic acid, (7) epicatechin, (8) vanillin, (9) catechin gallate, (10) p-coumaric acid, (11) ferulic acid, (12) ellagic acid derivative, (13) quercetin. BNB, Brazil nut beverage; PAS, pasteurized beverage (63 °C, 20 min); T1, HPH-treated to 50 MPa/75 °C; T2, HPH-treated to 150 MPa/55 °C; T3, HPH-treated to 180 MPa/75 °C.

The phenolic compounds of the control and processed Brazil nut beverages were identified and quantified via HPLC-UV/Vis-MS. The phenolic compounds in Brazil nut beverage were reported in a previous study [5], which identified the 13 most abundant phenolic compounds at 280 nm—mainly hydroxybenzoic acids—and at 320 nm for flavanols as well as at 380 nm for flavonoids, Table S3, Supplementary Materials. Figure 2 shows the HPLC C18 chromatogram of phenolic compounds from Brazil nut beverage detected at 280 nm. Phenolic quantification of individual compounds of BNB showed the predominance of flavonoids (57.9 mg/L), followed by phenolic acids (26.7 mg/L) and phenolic aldehydes (0.8 mg/L), Table 4. The most abundant individual phenolic compounds, including derivative compounds, were catechin, epicatechin, catechin gallate, and ellagic acid, with a concentration range of 13 to 17 mg/L. Previous studies also observed catechin and its derivatives as one of the main flavonoids present in BN and its beverage [5,38]. Total phenols of BNB were 85.3 mg/L, consistent with the TPC value (98.2 mg GAE/L) obtained using the Folin–Ciocalteu method. These values are similar to previous reports for Brazil nut beverage (71 mg GAE/L) [4], which were higher than that of almond milk (1.24 mg GAE/L) and similar to that of soymilk (88 mg GAE/L) [39]. These values can change for several reasons, such as plant variety, beverage formulation, and production process.

Table 4 shows the total phenolic contents of the different samples of processed BNBs, noting a reduction between 24 to 30% from untreated BNB control sample (85.3 mg/L) at day 0 (just after processing). No significant differences (*p* ≤ 0.05) were observed between the HPH- and pasteurization-processed BNBs on total phenolics, which ranged from 60 to 65 mg/L. These results are consistent with previously reported studies on soy milk, which showed a reduction in TPC between 7.5 and 42.5% depending of the characteristics of the thermal process and the soybean varieties [40]; in almond milk, there was a 5% reduction in TPC when pasteurized at 90 °C at 60 s [15]. Reported studies of HPH process on the TPC of plant-based milks are scarce, but it is known that mechanical stresses and hydrodynamic cavitation of the HPH process favors the release of intracellular cell wall components, enhancing the extraction of phenolic compounds [41], as was seen in grape and orange juice subjected to 250 MPa [42]. However, an opposite effect (similar to that observed in the current study) was reported in HPH-treated apple juice at 250 MPa [43] and mandarin juice at 20 MPa [42], with a reduction in TPC attributable to the forces and temperature stresses created in the homogenizing valve. In addition to the combination of pressure and temperature, the number of cycles applied can influence the level of phenolic compounds, as seen in HPH-treated strawberry nectar [44].

At the level of individual phenolic compounds, as shown in Table 4, the phenolic compounds most sensitive to the processing (HPH process and pasteurization) were gallic acid, catechin, epicatechin, catechin gallate, and ellagic acid in just-processed BNB (at day 0). In the rest of the remaining phenolic compounds, the effect of the HPH process was negligible or not significant (*p* ≥ 0.05). Consequently, the total phenolic acids (15.4 to 16.9 mg/L) and total flavonoids (43.5 to 46.9 mg/L) suffered a significant reduction due to the treatments applied. Previous studies on soy milk subjected to a thermal process observed a complex variation in the phenolic profile. Some suffered reduction, and others increased, but the total phenolic acid and total flavonoid contents were not modified [45]. In another report on almond milk, the thermal process produced a reduction in total flavonoids [14]. In the HPH process of fruit juices, a variable effect on individual phenols was also reported [46], revealing the specific characteristics of the release and thermal stability of each phenolic compound. The results of the present study and the data of previously published reports are consistent with the claim that HPH processing affects the stability of phenolic compounds depending on several aspects, such as phenolic profile, matrix type, and the intensity of the process (pressure, temperature, cycles) [47].

The stability of the individual phenolic compounds of HPH-processed BNB during cold storage was also studied. An increase in total phenols was observed in all beverages (treated with HPH and pasteurization), i.e., from 9 to 17% at day 9 and from 6.8 to 15% at day 21, were observed for total phenolic compounds, which was also verified with the obtained values of TPC analyzed using spectrometry. These increases were noticeable in the flavonoid compounds and to a lesser extent in phenolic acids. The most abundant individual phenolics that showed an increase during the cold storage of BNB were catechin, epicatechin, catechin gallate, and ellagic acid. A similar effect on phenolic compounds was reported for soy milk treated in HPH and UHT, with an increase in total isoflavones in the second month of storage at 20 °C [46]. This fact could be attributed to the better extraction of phenolic compounds linked to the cellular membranes, which were damaged by the HPH process. During cold storage, an increase in TPC content was noted in several commercial fruit beverages during cold storage for 20 days. This was attributed to reactions between oxidized polyphenols and new compounds of antioxidant character formed during storage [48]. However, a contrasting result was observed in pasteurized almond milk (at 90 °C, 60 s) [14] and HPH-treated soy milk (at 200 MPa, 55 to 75 °C) [49], for which a reduction in TPC and flavonoid contents of around 10% after storage for about 24 days at 4 °C took place. Phenolic degradation in vegetable beverages during storage is associated with various factors—such as food matrix, the content of dissolved oxygen, the storage temperature, and the enzymatic residual activity—able to promote the oxidation and polymerization of phenolic compounds [50]. The phenolic stability during cold storage of the Brazil nut beverages suggested that a possible inactivation of enzymes related to the degradation of phenolic compounds as polyphenoloxidase and peroxidase was produced by HPH and pasteurization processes, and for this reason, a minimal degradation of phenolic compounds took place.

### 2.4. Effect of HPH Process and Pasteurization on the Antioxidant Capacity

**Figure 3:**
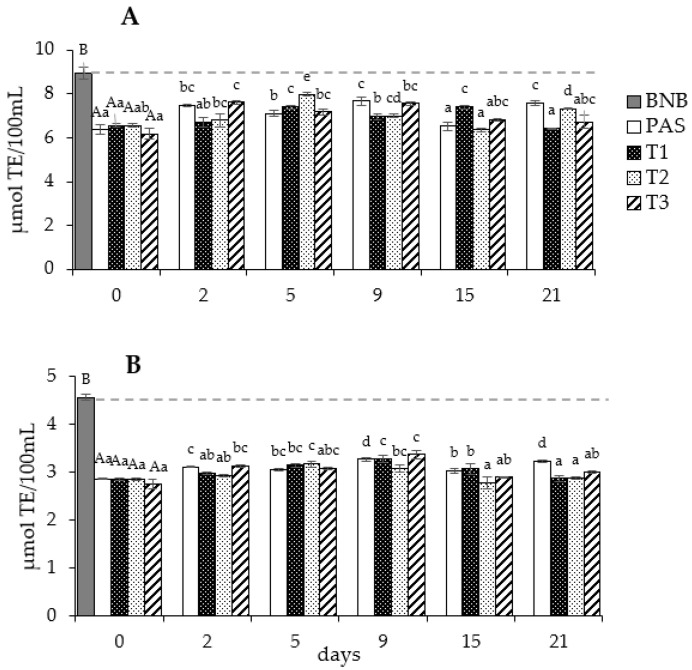
Antioxidant capacity ^1^ according to the (**A**) DPPH method and (**B**) ABTS method of Brazil nut beverage treated with HPH and pasteurization under cold storage for 21 days. ^1^ Data are expressed as mean ± standard deviation (*n* = 3). Different superscript capital letters indicate statistically significant differences (*p* ≤ 0.05) between treatments on day 0. Different superscript lowercase letters indicate statistically significant differences (*p* ≤ 0.05) between conservation days within the same treatment.

The effects of the processing treatments (HPH and pasteurization) during cold storage on the antioxidant capacity (AC) of Brazil nut beverages are shown in Figure 3 and Table S3, Supplementary Materials. On day 0, a significant (*p* ≤ 0.05) decrease in antioxidant capacity of 30 to 37% as analyzed using the ABTS and DPPH methods was observed in BN beverage BNBs just after processing (HPH and pasteurization). However, there were differences between the applied HPH treatments, with a range between 6.18 to 6.36 µmol TE/100 mL for AC with the DPPH method and between 2.76 and 2.87 µmol TE/100 mL for AC with the ABTS method. The antioxidant capacity reduction could be associated with the degradation of the phenol compounds shown above. Studies on HPH’s effect on the AC of other plant-based beverages are scarce, and they were mainly reported only for processed fruit juices; authors reported that HPH processing produced a better preservation of their antioxidant capacity (AC) than the thermal process [6]. However, an opposite effect or no differences were also reported between HPH and pasteurization in smoothies (milk and fruit juices) [43,51,52]. These changes could be attributed to the fact that certain antioxidant compounds are degraded during processing, but others could increase via antioxidant compound release after cell wall disruption caused by HPH and thermal processing.

The antioxidant capacity (AC) of processed vegetable beverages tend to decrease during cold storage due to the oxidation of antioxidant compounds, mainly attributed to the residual activity of oxidative enzymes, such as polyphenol oxidase, peroxidase, and lipoxygenase [53]. According to Figure 3, after the processing of the BNBs, the levels of AC remained stable until day 21, with no differences between HPH and pasteurization processes. These data correlated with their phenolic contents as explained before. The stability of AC values and the phenolic compound’s content may be associated with microbial stability, with the probable strong inactivation of the oxidative enzymes by HPH, and with the pasteurization processes. The observed AC stability is consistent with the data reported by previously published studies about vegetable beverages cold stored for short and medium periods [14,54,55]. Soy-based drinks generally keep their TPC content and AC stable, but a pronounced change is noticeable after only 9 months of storage [54]. In fruit beverages, after pasteurization (90 °C, 5 min), up to 3 h per month of storage with no modification of AC is reported [55]. Almond milk stored up to 28 days after thermal processing had a decrease in AC of about 15% [14]. An additional factor that could have contributed to the AC stability of Brazil nut beverages is the protective effect of partial protein denaturation, as observed in soy milks for protein-bound isoflavones [53]. In vegetable beverages, phenolic compounds and vitamin C are the main compounds associated with the antioxidant capacity of the beverage during storage, but complex reactions may occur in the long term—such as enzymatic polymerization of phenolic compounds—that increase the total antioxidant capacity or the presence of antioxidant antagonism and interaction reactions with opposite effects [48].

### 2.5. Effect of HPH Process and Pasteurization on the Minerals

Essential minerals are necessary to build the tissues of the human body and to maintain physiological functions and biochemical metabolism. These mineral elements cannot be synthesized in the body and must be supplied by foods [56]; Brazil nut is considered an appreciable source [2]. Table 5 shows the mineral contents of untreated Brazil nut beverage (BNB) and HPH- and pasteurization-processed BNBs during cold storage. Regarding the BNB macrominerals, potassium (K) (55.5 mg/100 mL) was the mineral which predominated in BNB beverages, followed by phosphorous (P) (22.1 mg/100 mL) and calcium (Ca) (5.2 mg/100 mL). For microminerals, the order of abundance was: copper (Cu) (167.3 µg/100 mL), zinc (Zn) (58.3 µg/100 mL), iron (Fe) (54.5 µg/100 mL), manganese (Mn) (35.1 µg/100 mL), and selenium (Se) (11.7 µg /100 mL). These results are consistent with previous reports on the same beverage [5,57]. Considering the recommended intake for adults ≥ 18 years stated by the European Food Safety Authority (EFSA) [58], one cup (~240 mL) of BNB would have a contribution of: Se: 40%, Cu: 27%, P: 10%, K: 4%, and Mn: 3%. The remarkable levels of Se and Cu in BNB are beneficial because they are central components of many enzymes and proteins and due to their critical role in many metabolic and cellular functions [58].

The effects of HPH and pasteurization treatments on mineral concentration were evaluated on day 0 (Table 5). All beverages, untreated and treated (HPH and pasteurization process), showed no statistical differences (*p* ≥ 0.05) in the content of the analyzed minerals (Table 5). For macrominerals, the ranges of values were P (19.1 control to 22.2 mg/100 mL processed), K (45.3 control to 56.8 mg/100 mL processed), and Ca (3.9 control to 5.2 mg/100 mL processed). For microminerals, the ranges were Cu (158.8 control to 170 µg/100 mL processed), Zn (52.8 control to 60.9 µg/100 mL processed), Fe (54.5 control to 61.8 µg/100 mL processed), Mn (29.8 control to 36.7 µg/100 mL processed), and Se (11.3 control to 14 µg/100 mL processed). In addition, the mineral profiles of each beverage were stable during cold storage for up to 21 days. Published studies of processing/storage’s effect on the mineral content of the plant-based beverages are scarce, but there are records of some analogous beverages that showed similar results. In milk smoothies and soy smoothies, neither HPP nor thermal processing significantly affected mineral content during cold storage for 45 days [57]. In maize beverage treated at various intensities of thermal processing (temperature from 63 to 85 °C and time from 5 to 30 min), statistical differences in minerals were also not observed [58]. The observed effects of HPH and pasteurization processes obtained in the present study are consistent with the statement that the change of mineral concentration in processed foods are generally caused by environmental factors, removed from the food by physical processes, or added (as additives or traces of the instruments). The loss of minerals from the point of view of bioaccessibility can occur through interaction with other substances—such as oxalic acid and phytic acid—that are widely present in plant foods, forming insoluble complexes that reduce absorption and digestibility [59].

## 3. Materials and Methods

### 3.1. Plant Material

Brazil nuts—dry seed, shelled (without woody tegument)—were purchased from a local market in Madrid (Spain). The BN composition in wet weight was wet 2.2% total lipids 66.1%, proteins 17.3%, carbohydrates 10.9%, and ash 3.4%. Vacuum-packaged Brazil nuts were stored under refrigeration at 5 °C until its processing and assays.

### 3.2. Solvents, Reagents, and Standards

Methanol (99.8% LC-MS) was purchased from VWR International (Barcelona, Spain). Ultra-pure water (Mili-Q) was obtained from a Millipak^®^ Express 40 system (Merck Millipore, Darmstadt, Germany). Acetone, sodium carbonate, n-hexane, formic acid, and chloroform solvents were purchased from VWR (Barcelona, Spain). Potassium peroxydisulfate (K_2_S_2_O_8_), potassium phosphate (KH_2_PO_4_), sodium phosphate (NaH_2_PO_4_ × 2H_2_O), Trolox (6-hydroxy-2,5,7,8-tetramethylchroman-2-carboxylic acid), N, O-bis (trimethylsilyl) trifluoroacetamide (BSTFA), Folin–Ciocalteu reagent, 2,2-diphenyl-1-picrylhydrazyl (DPPH), 2,20-azino-bis(3-ethylbenzthiazoline-6-sulfonic acid) (ABTS), and standards for GC analysis (oleic acid, DL-α-tocopherol, squalene, and β-sitosterol) and for HPLC analysis (catechin, epicatechin, vanillic acid, protocatechuic acid, quercetin, vanillin, gallic acid, and hydroxybenzoic acid) were purchased from Sigma-Aldrich (St. Louis, MO, USA).

### 3.3. Production of Brazil Nut Beverage

Production of Brazil nut beverage (BNB) was performed, taking as a reference the method reported by Vasquez-Rojas et al. [4] with some modifications. Briefly, Brazil nuts were previously ground and then homogenized at 10,000 rpm in a T-25 Ultraturrax (IKA-Werke GmbH & Co. KG. Staufen, Germany) with water at 75 °C in a 7:1 ratio (water: raw material, *v*/*w*) for five minutes. Then, this solution was immediately filtered with filter cloth (≤1 mm) to obtain the hot aqueous extract, which was subsequently cooled to 5 °C in an ice bath. The beverage was kept at rest for 15 h at 5 °C to remove the cream and sediment that formed.

### 3.4. HPH Treatment and Pasteurization of Brazil Nut Beverage

These conditions were selected in light of the previously published *E.coli* inactivation study [10]. Briefly, as the first part of the study of the stabilization of BNB, the level of *E. coli* reduction (inoculated in the BNB) by HPH and thermal processes was evaluated. HPH treatments were applied using a high-pressure homogenizer (Panda Plus 2000, GEA Niro Soavi, Parma, Italy) with a maximum capacity of 200 MPa. Pressures of 50, 100, 150, and 180 MPa and inlet temperatures of 25, 55, and 75 °C were tested. The inlet temperatures were obtained using an external hot water bath, and the HPH-treated beverage was immediately collected in sterile falcon tubes and cooled to 5 °C using an ice bath. As a control of traditional thermal treatment, pasteurization was conducted at 63 °C for 20 min, with a posterior cooling of the beverage to 5 °C using the ice bath, which was then collected in sterile falcon tubes. In the second part of the study [10], the best HPH conditions for *E. coli* reduction (≥5 log CFU/mL) were selected to study the stability of the beverage under cold storage, up to 21 days at 5 °C, evaluating the physical, microbiological, physicochemical, and sensory aspects; the pasteurized beverage was used as a control [10].

The same selected HPH treatments used in this previous study [10] were applied in the present work; the treatments consisted of three combinations of pressure and temperature, as follows: T1: 50 MPa/75 °C, T2: 150 MPa/55 °C, and T3: 180 MPa/75 °C. The inlet temperatures were obtained using an external hot water bath, and the homogenized beverage was collected in sterile glass containers and immediately cooled to 5 °C in an ice bath. Inlet temperature, temperature after the high-pressure valve, and outlet temperature were monitored. The BNB pasteurized at 63 °C for 20 min was used as a control sample.

### 3.5. Total Protein, Total Carbohydrates, and Ash Determinations

The total nitrogen content was analyzed with the Kjeldahl method, using a nitrogen-to-protein conversion factor of 6.25 with a Kjeltec 2300 analyzer (FOSS Tecator Technology, Parma, Italy) according to AOAC (2005) [60]. Total carbohydrates were analyzed according to the phenol–sulfuric acid method [61], and the ash was determined by the reported gravimetric method [62].

### 3.6. Determination of Minor Lipid Compounds

#### 3.6.1. Lipid Extraction

The lipids in the samples were extracted using the method of Folch, Lees, and Sloane Stanley [63] with some modification. Briefly, 5 g of sample was mixed with 20 mL of chloroform–methanol (2:1, *v*/*v*) to be homogenized in a T-25 Ultraturrax (IKA-Werke GmbH & Co., KG, Staufen, Germany) at 11,000 rpm for 3 min and then centrifuged at 1050× *g* for 10 min. The bottom organic phase was collected and filtered with paper and dried with anhydrous sodium sulphate. The lipid solution obtained was dried on a rotary evaporator under vacuum at 40 °C and held at −20 °C until analysis.

#### 3.6.2. Fatty Acids Analysis

The analysis was performed following the procedure reported by Vasquez et al. [64]. The fatty acids (FA) profile of lipid samples was determined after the derivatization of fatty acid methyl esters (FAMEs). Briefly, 20 mg oil was mixed with 0.5 mL chloroform/methanol (2:1; *v*/*v*) and 1 mL of 0.1 M NaOH in methanol. This mixture was heated at 60 °C for 30 min, then 0.2 mL of distilled water was added. The FAMEs formed were extracted with 1 mL of hexane by vortexing. They were then decanted, and the upper phase was collected. The hexane was removed under a nitrogen atmosphere. The obtained FAMEs were dissolved in hexane to a concentration of 1 mg/mL for each sample and injected into a gas chromatograph Agilent 7890 A (Agilent Technologies, Santa Clara, CA, USA) equipped with a split–splitless injector and an autosampler, a flame ionization detector, and a triple-axis mass spectrometer detector. FAMEs were separated using an HP-5MS column (30 m length, 0.25 mm internal diameter, and 0.25 µm film thickness). Helium was used as a carrier gas at 2 mL/min. The injection temperature was 260 °C, splitless mode, 1 µL, and the mass spectrometer ion source and interface temperatures were 230 and 280 °C, respectively. The chromatographic analysis started at 50 °C and increased at a speed of 20 °C/min until it reached 210 °C. It remained at that temperature for 18 min. Then, it increased to 230 °C and remained there for 13 min. The running time was 40 min. The mass spectra were obtained through electronic ionizing impact at 70 eV. The scan rate was 1.6 scans/s at a mass range of 30–700 amu. The identification of fatty acids was performed using the NIST MS Data library. The area under each FA peak in relation to the total area of all FA peaks was used for relative quantification and expressed as a percentage of FA.

#### 3.6.3. Free Fatty Acids, Tocopherol, Phytosterol, and Squalene Analysis

The procedure reported by Vasquez et al. [64] was followed. The lipid extracts were derivatized using N and O-bis(trimethylsilyl)trifluoroacetamide (BSTFA). Briefly, 5 mg of the oil was mixed with 1 mL of the derivatizing agent and then subjected to 75 °C for 1 h, shaking every 15 min. Finally, it was injected into the previously described chromatography equipment. The separation procedure was started at 50 °C, was held for 3 min—increasing to 310 °C at a rate of 15 °C/min—and was held for 25 min. For quantification, calibration curves were obtained from the standards of α-tocopherol, squalene, oleic acid, and β-sitosterol, which were also previously derivatized.

### 3.7. Determination of Phenolic Compounds

#### 3.7.1. Extraction

Phenolic compounds were extracted using the method reported by John and Sha-hidi [65], with some modifications. Each sample was previously freeze-dried (Lyobeta-15, Azbil Telstar, S.L., Terrasa, Spain) and then defatted with hexane (1:5 *w*/*v*, ×2), homogenized for 5 min, and filtered through filter paper. The solid residue (defatted) was dried at room temperature for 12 h in the dark. It was then extracted with acetone in a ratio of 1:15 (*w*/*v*), homogenized using a T-25 Ultraturrax (IKA-Werke GmbH & Co., KG, Staufen, Germany) at 11,000 rpm for 5 min, and later submitted to reflux in a water bath at 60 °C for 40 min with magnetic stirring. The resulting slurry was centrifuged at 15,000× *g* for 10 min, and the supernatant was collected. The sediment was re-extracted under the same conditions. Then, the supernatants were combined, and the solvent was removed by vacuum at 40 °C. The extract containing the phenolic compounds was stored at −20 °C until its use for the analysis of total phenolics and antioxidant activity.

#### 3.7.2. Total Phenolic Content

The total phenolics in the samples (phenolic extracts) were analyzed spectrophotometrically with Folin–Ciocalteu reagent according to the method of Cano et al. [66], with some modifications. In a 96-microwell plate, the reactants were placed in the following order: 20 μL samples (extracts, standard, and blank) were reacted with 100 μL Folin–Ciocalteu reagent (10%, *v*/*v*) and then alkalized with 80 μL of Na_2_CO_3_ 7.5% (*w*/*v*). The mixture was stirred and kept in darkness for one hour at 20 °C. The reading was taken spectrophotometrically at 756 nm (Bio-Tek instruments, Winooski, VT, USA). Gallic acid was used as the reference standard to elaborate the calibration curve with concentrations in the range of 50 to 500 μg/mL. The results were expressed as milligrams of gallic acid equivalents (GAE)/L.

#### 3.7.3. Analysis of Individual Phenolic Compounds

Phenolic extracts were dissolved with methanol/water (2:1, *v*/*v*) and filtered with 0.45 µm syringe filters before injection into HPLC UV/Vis. Chromatographic analyses were performed according to the protocol reported by García-Cayuela et al. [67] using a 1200 Series Agilent HPLC System (Agilent Technologies, Santa Clara, CA, USA) with a reverse phase C18 column (Zorbax SB-C18, 250 × 4.6 mm, 5 μm; Agilent) maintained at 20 °C. Elution solvent A consisted of 1% formic acid (*v*/*v*) in water, while solvent B was a mixture of methanol and formic acid (1%, *v*/*v*). Separation was achieved using an initial solvent composition of 15% (B) for 15 min, increased to 25% (B) after 10 min, ramped to 50% (B) after another 10 min, increased to 75% (B) after another 15 min. This was followed by a decreased period of 15% (B) for 5 min prior to isocratic re-equilibration at 15% (B) for 10 min. The flow rate was fixed at 0.8 mL/min, and the injection volume was 20 μL. The UV/Vis photodiode array detector was set at three wavelengths (280, 320, 380 nm) for monitoring different phenolic chemical families simultaneously.

The HPLC was coupled to a mass spectrometry detector (LCMS SQ 6120, Agilent, Santa Clara, CA, USA) with an electrospray ionization (ESI) source operating in positive ion mode. The drying gas was nitrogen at 3 L/min at 137.9 KPa. The nebulizer temperature was 300 °C, and the capillary had 3500 V potential. The collision gas was helium, and the fragmentation amplitude was 70 V. Spectra recorded *m*/*z* from 100–1000. Further mass spectrometry analyses were performed using maXis II LC-QTOF equipment (Bruker Daltonics, Bremen, Germany) with an ESI source and the same chromatographic conditions. The ESI-QTOF detector worked in positive ion mode and recorded spectra *m*/*z* from 50–3000. The operation conditions were 300 °C, capillary voltage 3500 V, charging voltage 2000 V, nebulizer 2.0 bar, and dry gas at 6 L/min. MS/MS analysis used the bbCID (broadband collision-induced dissociation) method at 30 eV. Phenolic compounds were identified according to their retention times, UV/Vis, and mass spectral data compared to those of commercial standards. Quantitation of the phenolic compounds was determined using the calibration curves of the corresponding standards using a diode array detector at λ = 280, 320, and 370 nm, depending on the phenolic chemical identity as previously reported by García-Cayuela et al. [67].

### 3.8. Antioxidant Capacity

#### 3.8.1. DPPH Assay

The antioxidant capacity was determined by the DPPH (2,2-diphenyl-1-picrylhy-drazyl) radical scavenging method according to Vasquez-Rojas et al. [5]. In a 96-well microplate, the reaction was carried out as follows: a 50 µL aliquot of the sample (phenolic extract), previously diluted with methanolic solution (70%), was mixed with 250 µL of DPPH (0.5 mM), and after 1 h in the dark, the absorbance was measured at 517 nm using spectrophotometric equipment (Bio-Tek instruments, Winooski, VT, USA). The reference standard used for the calibration curve was Trolox (6-hydroxy-2,5,7,8-tetramethylchroman-2-carboxylic acid) at different concentrations (100 to 800 µM/mL). The antioxidant capacity was expressed as µmol Trolox equivalent (TE)/100 mL.

#### 3.8.2. TEAC Assay

The procedure was according to Vasquez-Rojas et al. [5]. The radicals ABTS+ were generated through 44 µL potassium peroxydisulfate (K_2_S_2_O_8_) 140 mM and 2.5 mL ABTS 7 mM. A part of this ABTS stock solution was diluted with phosphate-buffered saline (PBS, 75 mM, pH 7.4) consisting of potassium phosphate (KH_2_PO_4_) and sodium phosphate (NaH_2_PO_4_) until an optical density of 0.70 ± 0.02 at 734 nm was achieved. TE solutions (10 to 100 µM) were used for calibration. The reaction was initiated by mixing 30 µL of the sample extract, or standard, with 200 µL of ABTS+, then left to rest 40 min in the dark. The absorbance was measured spectrophotometrically at 734 nm. Antioxidant capacity was expressed as µmol TE/100 mL.

### 3.9. Minerals Analysis

Around 1.5 mL of the samples was digested via microwave-assisted acid digestion with 4 mL of HNO_3_. The reactors were introduced to the UltraWAVE digestion system (Milestone, Sorisole, Italy) and heated up to 240 °C for 20 min with a base working pressure of 40 bars. Qualitative and quantitative TXRF analysis of the samples were performed using a benchtop TXRF spectrometer (S2 PICOFOX, Bruker Nano GmbH, Berlin, Germany) equipped with a molybdenum X-ray source working at 50 kV and 600 µA, a multilayer monochromator with 80% of reflectivity at 17.5 keV (Mo Ka), a XFlash SDD detector with an effective area of 30 mm^2^, and an energy resolution better than 150 eV for 5.9 keV (Mn Ka). The acquisition time for qualitative analysis was 300 s, and that for the quantitative analysis was 1000 s. Cobalt (Co) was chosen as the internal standard for quantification mainly because this element was not present in the samples. The Spectra 7.5.3 software from Bruker was used for the control, acquisition, deconvolution, and integration of all analyzed samples.

### 3.10. Statistical Analyses

Analyses were in triplicate and expressed as mean and standard deviation. The obtained results were evaluated with variance analysis two-way ANOVA, and differences between means were evaluated using Tukey’s test. The significant statistical differences were calculated at a *p* < 0.05 level. The statistical software employed was IBM SPSS Statistic 20.0.

## 4. Conclusions

The effect of high-pressure homogenization (HPH) compared to thermal processing (pasteurization) on phytochemicals, nutrients, and antioxidant capacity was studied. There were no relevant changes in the fatty acid profile or free fatty acids after both processing treatments and during cold storage. Only losses of on squalene (24%) and γ-tocopherol (32%) were observed, which could be associated with the enzyme inactivation produced by processing. The treatments (HPH or pasteurization) affected the phenol profile of BNBs. Total phenols were reduced in a range of 24 to 30% compared to untreated BNB (85.3 mg/L) due to HPH and pasteurization treatments. This fact influenced the antioxidant capacity of the processed BNBs. The individual phenolic compounds most sensitive to the processing were gallic acid, catechin, epicatechin, catechin gallate, and ellagic acid. During cold storage, any significant reduction in total phenols was observed in all beverages. Total protein and essential minerals were not significantly modified by any treatments during cold storage. Therefore, the HPH process is an alternative technique to thermal treatment to obtain a stable BNB. The replacement of traditional thermal treatment can represent an advantage for the industry since this HPH is a cold technology with a lower impact on the environment and greater sustainability, saving energy, time, and additional costs. In the present work, HPH technology produced a safe and fresh-like BNB with very low changes in composition. These results help to identify technological alternatives for processing vegetable beverages, and their influence on the phytochemical composition focused on the production of functional foods.

## Figures and Tables

**Table 1:** Total protein content (g/100 mL) ^1^ of Brazil nut beverages treated with HPH and pasteurization during cold storage at 5 °C.

Days	BNB	PAS	T1	T2	T3
0	1.09 ± 0.04 ^A^	1.09 ± 0.04 ^Aa^	1.06 ± 0.00 ^Aa^	1.08 ± 0.03 ^Aa^	1.09 ± 0.01 ^Aa^
2	N/A	1.05 ± 0.05 ^a^	1.08 ± 0.05 ^a^	1.12 ± 0.01 ^a^	1.07 ± 0.07 ^a^
5	N/A	1.10 ± 0.10 ^a^	1.03 ± 0.04 ^a^	1.05 ± 0.09 ^a^	1.08 ± 0.03 ^a^
9	N/A	1.13 ± 0.09 ^a^	1.04 ± 0.09 ^a^	1.08 ± 0.16 ^a^	1.11 ± 0.01 ^a^
15	N/A	1.05 ± 0.07 ^a^	1.10 ± 0.13 ^a^	1.06 ± 0.07 ^a^	1.07 ± 0.11 ^a^
21	N/A	1.14 ± 0.02 ^a^	1.09 ± 0.14 ^a^	1.02 ± 0.13 ^a^	1.09 ± 0.05 ^a^

^1^ Data are expressed as mean ± standard deviation (*n* = 3). N/A indicates samples not analyzed. Different superscript capital letters indicate statistically significant differences (*p* ≤ 0.05) between treatments on day 0. Different superscript lowercase letters indicate statistically significant differences (*p* ≤ 0.05) between conservation days within the same treatment.

**Table 2:** Fatty acid contents ^1^ of Brazil nut beverages treated with HPH and pasteurization during cold storage (5 °C).

Fatty Acid (%)	Day	BNB	PAS	T1	T2	T3
Palmitic acid	0	14.9 ± 0.0 ^AB^	15.0 ± 0.0 ^Ba^	14.9 ± 0.0 ^Aba^	14.9 ± 0.0 ^Aba^	14.9 ± 0.0 ^Aa^
	9		16.6 ± 0.0 ^b^	16.3 ± 0.1 ^b^	16.3 ± 0.1 ^b^	16.3 ± 0.0 ^b^
	21		14.9 ± 0.1 ^a^	15.0 ± 0.0 ^a^	14.9 ± 0.0 ^a^	14.8 ± 0.0 ^a^
Oleic acid	0	40.4 ± 0.4 ^A^	40.7 ± 0.3 ^Aa^	40.6 ± 0.0 ^Ab^	40.8 ± 0.3 ^Ab^	40.5 ± 0.0 ^Aa^
	9		39.9 ± 0.3 ^a^	39.8 ± 0.1 ^a^	39.9 ± 0.1 ^a^	40.8 ± 1.1 ^a^
	21		40.5 ± 0.1 ^a^	40.4 ± 0.3 ^ab^	40.0 ± 0.3 ^ab^	40.4 ± 0.1 ^a^
Linoleic acid	0	34.3 ± 0.4 ^A^	34.0 ± 0.3 ^Aa^	34.1 ± 0.1 ^Aa^	33.8 ± 0.2 ^Aa^	34.4 ± 0.1 ^Aa^
	9		33.4 ± 0.3 ^a^	33.8 ± 0.2 ^a^	33.8 ± 0.0 ^a^	32.8 ± 1.0 ^a^
	21		34.2 ± 0.3 ^a^	34.3 ± 0.3 ^a^	34.7 ± 0.3 ^a^	34.5 ± 0.1 ^a^
Stearic acid	0	10.4 ± 0.0 ^A^	10.4 ± 0.0 ^Aa^	10.4 ± 0.0 ^Ab^	10.4 ± 0.0 ^Ab^	10.3 ± 0.0 ^Ab^
	9		10.0 ± 0.0 ^b^	10.1 ± 0.0 ^a^	10.0 ± 0.0 ^a^	10.1 ± 0.0 ^a^
	21		10.4 ± 0.1 ^b^	10.4 ± 0.0 ^b^	10.4 ± 0.1 ^b^	10.3 ± 0.0 ^b^

^1^ Data are expressed as mean ± standard deviation (*n* = 3). Different superscript capital letters indicate statistically significant differences (*p* ≤ 0.05) between treatments on day 0. Different superscript lowercase letters indicate statistically significant differences (*p* ≤ 0.05) between conservation days within the same treatment.

**Table 3:** Minor lipid content ^1^ (mg/L) of Brazil nut beverage after HPH and pasteurization process and cold storage (5 °C).

Compound mg/L	Day	BNB (Control)	PAS	T1	T2	T3
Squalene	0	70.3 ± 4.5 ^B^	54.3 ± 3.7 ^Aa^	53.0 ± 1.6 ^Aa^	51.7 ± 3.3 ^Aa^	53.4 ± 2.7 ^Ab^
	9		55.3 ± 3.4 ^a^	51.2 ± 0.5 ^a^	49.0 ± 4.1 ^a^	45.8 ± 0.1 ^a^
	21		57.5 ± 2.2 ^a^	54.3 ± 3.6 ^a^	51.0 ± 1.4 ^a^	49.3 ± 0.7 ^ab^
Tocopherol	0	10.4 ± 0.5 ^B^	7.4 ± 0.3 ^Aa^	7.5 ± 0.1 ^Ab^	7.1 ± 0.2 ^Aa^	6.7 ± 0.2 ^Aa^
	9		7.4 ± 0.4 ^a^	6.9 ± 0.0 ^a^	6.9 ± 0.3 ^a^	6.4 ± 0.0 ^a^
	21		7.9 ± 0.5 ^a^	7.4 ± 0.1 ^b^	7.1 ± 0.2 ^a^	6.8 ± 0.4 ^a^
β- Sitosterol	0	35.6 ± 0.9 ^A^	35.4 ± 0.8 ^Ab^	34.3 ± 1.8 ^Aa^	33.9 ± 1.4 ^Aa^	33.7 ± 0.1 ^Ab^
	9		29.4 ± 1.4 ^a^	28.6 ± 0.5 ^a^	30.4 ± 2.2 ^a^	29.4 ± 0.2 ^a^
	21		33.2 ± 1.3 ^ab^	32.1 ± 1.7 ^a^	30.3 ± 1.7 ^a^	30.6 ± 0.6 ^a^

^1^ Data are expressed as mean ± standard deviation (*n* = 2). Different superscript capital letters indicate statistically significant differences (*p* ≤ 0.05) between treatments on day 0. Different superscript lowercase letters indicate statistically significant differences (*p* ≤ 0.05) between conservation days within the same treatment.

**Table 4:** Individual phenolic compound content ^1^ (mg/L) of Brazil nut beverage treated with HPH and pasteurization under cold storage for 21 days at 5 °C.

Peak	Phenolic Compound	RT (min)	BNB	PAS			T1			T2			T3		
			Day 0	Day 0	Day 9	Day 21	Day 0	Day 9	Day 21	Day 0	Day 9	Day 21	Day 0	Day 9	Day 21
1	Gallic acid	5.7	2.5 ± 0.0 ^E^	1.9 ± 0.0 ^Da^	1.9 ± 0.0 ^a^	1.8 ± 0.0 ^a^	1.6 ± 0.0 ^Ba^	1.8 ± 0.0 ^b^	1.7 ± 0.1 ^ab^	1.7 ± 0.0 ^Cb^	1.7 ± 0.0 ^b^	1.5 ± 0.0 ^a^	1.5 ± 0.0 ^Aa^	1.6 ± 0.0 ^b^	1.6 ± 0.0 ^b^
2	Gallic acid derivative	6.2	2.5 ± 0.0 ^C^	1.5 ± 0.0 ^Aa^	1.7 ± 0.0 ^b^	1.8 ± 0.0 ^c^	1.6 ± 0.0 ^Ba^	1.8 ± 0.0 ^b^	1.6 ± 0.0 ^a^	1.6 ± 0.0 ^Bb^	1.6 ± 0.0 ^b^	1.5 ± 0.0 ^a^	1.4 ± 0.0 ^Aa^	1.5 ± 0.0 ^b^	1.5 ± 0.0 ^b^
3	Catechin	20.90	16.4 ± 0.5 ^B^	16.4 ± 0.1 ^ABa^	18.2 ± 0.2 ^b^	18.2 ± 0.3 ^b^	15.7 ± 0.1 ^ABa^	18.2 ± 0.1 ^b^	17.3 ± 0.4 ^b^	15.5 ± 0.7 ^ABa^	16.9 ± 0.3 ^a^	16.1 ± 0.2 ^a^	14.6 ± 0.5 ^Aa^	15.7 ± 0.2 ^a^	15.8 ± 0.9 ^a^
4	Catechin derivative	22.29	12.1 ± 0.7 ^B^	9.2 ± 0.2 ^Aa^	11.2 ± 0.2 ^b^	11.8 ± 0.6 ^b^	9.4 ± 0.7 ^Aa^	11.6 ± 0.4 ^b^	10.7 ± 0.2 ^ab^	9.5 ± 0.3 ^Aa^	10.9 ± 0.6 ^a^	10.8 ± 0.3 ^a^	8.6 ± 0.2 ^Aa^	9.8 ± 0.3 ^b^	9.9 ± 0.2 ^b^
5	4-hydroxybenzoic acid	26.92	0.9 ± 0.0 ^C^	0.9 ± 0.1 ^Cab^	0.8 ± 0.0 ^a^	1.0 ± 0.0 ^b^	0.8 ± 0.0 ^BCa^	0.9 ± 0.0 ^b^	0.9 ± 0.0 ^ab^	0.7 ± 0.0 ^ABa^	0.9 ± 0.0 ^b^	0.7 ± 0.0 ^a^	0.6 ± 0.0 ^Aa^	0.5 ± 0.0 ^a^	0.8 ± 0.0 ^b^
6	Vanillic acid	29.04	1.6 ± 0.1 ^B^	1.6 ± 0.1 ^Ba^	1.7 ± 0.1 ^a^	1.6 ± 0.0 ^a^	1.5 ± 0.1 ^Ba^	1.8 ± 0.1 ^a^	1.6 ± 0.0 ^a^	1.6 ± 0.0 ^Ba^	1.9 ± 0.1 ^b^	1.7 ± 0.1 ^ab^	1.1 ± 0.1 ^Aa^	1.7 ± 0.0 ^b^	1.7 ± 0.1 ^b^
7	Epicatechin	29.94	15.3 ± 0.8 ^B^	9.4 ± 0.0 ^Aa^	10.2 ± 0.1 ^b^	10.3 ± 0.2 ^b^	9.6 ± 0.0 ^Aa^	10.5 ± 0.1 ^c^	10.0 ± 0.0 ^b^	9.5 ± 0.3 ^Aa^	9.8 ± 0.3 ^a^	9.4 ± 0.1 ^a^	8.8 ± 0.1 ^Aa^	9.5 ± 0.1 ^b^	9.6 ± 0.1 ^b^
8	Vanillin	31.6	0.8 ± 0.0 ^AB^	0.8 ± 0.1 ^Ba^	0.9 ± 0.0 ^a^	0.9 ± 0.0 ^a^	0.7 ± 0.0 ^ABa^	0.9 ± 0.0 ^b^	0.7 ± 0.0 ^a^	0.7 ± 0.1 ^ABa^	0.8 ± 0.0 ^b^	0.8 ± 0.0 ^ab^	0.6 ± 0.0 ^Aa^	0.7 ± 0.0 ^ab^	0.8 ± 0.0 ^b^
9	Catechin gallate	32.9	13.0 ± 0.7 ^B^	11.1 ± 0.5 ^Aa^	13.4 ± 0.2 ^b^	13.3 ± 0.1 ^b^	10.3 ± 0.2 ^Aa^	12.4 ± 0.3 ^b^	11.6 ± 0.3 ^b^	10.5 ± 0.5 ^Aa^	12.1 ± 0.1 ^b^	11.8 ± 0.3 ^ab^	10.8 ± 0.3 ^Aa^	12.6 ± 0.2 ^b^	12.2 ± 0.1 ^b^
10	p-Coumaric acid	35.02	1.1 ± 0.0 ^C^	0.8 ± 0.0 ^Aba^	1.0 ± 0.0 ^a^	0.9 ± 0.0 ^a^	0.8 ± 0.0 ^Aa^	0.9 ± 0.0 ^b^	1.0 ± 0.0 ^b^	0.9 ± 0.1 ^Ba^	0.9 ± 0.0 ^a^	0.9 ± 0.0 ^a^	0.8 ± 0.0 ^ABab^	0.7 ± 0.0 ^a^	0.9 ± 0.0 ^b^
11	Ferulic acid	36.08	0.7 ± 0.0 ^B^	0.5 ± 0.0 ^Aa^	0.6 ± 0.0 ^b^	0.6 ± 0.0 ^ab^	0.5 ± 0.0 ^Aa^	0.7 ± 0.0 ^b^	0.5 ± 0.0 ^a^	0.5 ± 0.0 ^Aa^	0.6 ± 0.0 ^a^	0.6 ± 0.0 ^a^	0.4 ± 0.0 ^Aa^	0.5 ± 0.0 ^ab^	0.7 ± 0.0 ^b^
12	Ellagic acid derivative	37.60	17.3 ± 0.0 ^C^	9.8 ± 0.0 ^Ba^	11.0 ± 0.0 ^c^	10.8 ± 0.0 ^b^	9.7 ± 0.0 ^ABa^	10.8 ± 0.0 ^c^	10.0 ± 0.0 ^b^	9.7 ± 0.1 ^ABa^	10.2 ± 0.1 ^c^	10.0 ± 0.0 ^b^	9.6 ± 0.0 ^Aa^	10.2 ± 0.0 ^b^	10.1 ± 0.0 ^b^
13	Quercetin	38.62	1.0 ± 0.0 ^C^	0.9 ± 0.0 ^Ba^	1.2 ± 0.1 ^b^	1.1 ± 0.0 ^b^	0.9 ± 0.0 ^Ba^	1.2 ± 0.0 ^b^	1.1 ± 0.0 ^b^	0.9 ± 0.1 ^BCa^	1.1 ± 0.0 ^a^	1.0 ± 0.1 ^a^	0.7 ± 0.0 ^Aa^	1.1 ± 0.1 ^b^	1.1 ± 0.0 ^b^
	*Total Phenolic acids*		26.7 ± 0.1 ^C^	16.9 ± 0.2 ^Ba^	18.6 ± 0.1 ^b^	18.6 ± 0.1 ^b^	16.4 ± 0.2 ^Ba^	18.8 ± 0.0 ^b^	17.2 ± 0.0 ^b^	16.8 ± 0.1 ^Ba^	17.7 ± 0.1 ^b^	17.0 ± 0.1 ^a^	15.4 ± 0.0 ^Aa^	16.8 ± 0.0 ^b^	17.2 ± 0.1 ^c^
	*Total Phenolic aldehydes*		0.8 ± 0.0 ^AB^	0.8 ± 0.1 ^Ba^	0.9 ± 0.0 ^a^	0.9 ± 0.0 ^a^	0.7 ± 0.0 ^ABa^	0.9 ± 0.0 ^b^	0.7 ± 0.0 ^b^	0.7 ± 0.1 ^ABa^	0.8 ± 0.0 ^b^	0.8 ± 0.0 ^ab^	0.6 ± 0.0 ^Aa^	0.7 ± 0.0 ^ab^	0.8 ± 0.0 ^b^
	*Total Flavonoids*		57.9 ± 1.3 ^B^	46.9 ± 0.6 ^Aa^	54.3 ± 0.8 ^b^	54.7 ± 0.7 ^b^	45.9 ± 0.7 ^Aa^	53.9 ± 0.0 ^b^	50.6 ± 0.9 ^b^	46.0 ± 1.2 ^Aa^	50.9 ± 0.1 ^b^	50.1 ± 0.2 ^ab^	43.5 ± 1.1 ^Aa^	48.8 ± 0.3 ^b^	48.6 ± 1.2 ^b^
	*Total Phenolic comp.*		85.3 ± 1.5 ^C^	64.7 ± 0.8 ^Ba^	73.7 ± 0.7 ^b^	74.2 ± 0.5 ^b^	62.9 ± 0.6 ^ABa^	73.6 ± 0.1 ^b^	68.6 ± 0.8 ^b^	63.4 ± 1.3 ^ABa^	69.5 ± 0.0 ^b^	67.7 ± 0.3 ^ab^	59.5 ± 1.1 ^Aa^	66.2 ± 0.3 ^b^	66.6 ± 1.1 ^b^
	*TPC* (mg GAE/L)		98.2 ± 5 ^B^	61.0 ± 2.5 ^Aa^	73.3 ± 1.2 ^bc^	75.9 ± 2.2 ^c^	58.2 ± 2.4 ^Aa^	74.8 ± 3.4 ^d^	67.2 ± 0.2 ^bc^	60.8 ± 3.7 ^Aa^	69.2 ± 3.7 ^b^	66.1 ± 1 ^ab^	60.6 ± 2.3 ^Aa^	72.7 ± 1 ^b^	69.2 ± 5.1 ^b^

^1^ Data are expressed as mean ± standard deviation (*n* = 3). Different superscript capital letters indicate statistically significant differences (*p* ≤ 0.05) between treatments on day 0. Different superscript lowercase letters indicate statistically significant differences (*p* ≤ 0.05) between conservation days within the same treatment.

**Table 5:** Content of minerals ^1^ of Brazil nut beverage treated using HPH and pasteurization and under cold storage (5 °C) for 21 days.

Minerals	Day	BNB	PAS	T1	T2	T3
*Macrominerals* (mg/100 mL)						
P	0	22.1 ± 1.4 ^A^	22.2 ± 0.0 ^Aa^	21.1 ± 0.1 ^Aa^	19.1 ± 1.1 ^Aa^	21.2 ± 0.1 ^Aa^
	9	N/A	22.5 ± 0.8 ^a^	22.4 ± 0.5 ^a^	23.2 ± 0.1 ^b^	23.7 ± 0.5 ^b^
	21	N/A	22.8 ± 1.4 ^a^	22.4 ± 0.4 ^a^	22.3 ± 0.9 ^ab^	21.0 ± 0.4 ^a^
K	0	55.5 ± 5.5 ^A^	50.5 ± 2.1 ^Aa^	56.8 ± 3.0 ^Aa^	45.3 ± 2.4 ^Aa^	47.6 ± 1.3 ^Aa^
	9	N/A	45.3 ± 2.0 ^a^	49.1 ± 3.0 ^a^	47.0 ± 2.0 ^a^	48.5 ± 1.1 ^a^
	21	N/A	54.5 ± 3.5 ^a^	48.8 ± 2.7 ^a^	53.8 ± 1.6 ^a^	56.6 ± 2.8 ^b^
Ca	0	5.2 ± 0.0 ^C^	5.1 ± 0.1 ^BCa^	4.2 ± 0.3 ^ABa^	3.9 ± 0.2 ^Aa^	4.6 ± 0.3 ^ABCa^
	9	N/A	5.0 ± 0.0 ^a^	5.0 ± 0.4 ^a^	5.3 ± 0.2 ^b^	4.0 ± 0.5 ^a^
	21	N/A	5.8 ± 0.2 ^b^	5.1 ± 0.0 ^a^	5.4 ± 0.1 ^b^	4.8 ± 0.5 ^a^
*Microminerals* (µg/100 mL)						
Mn	0	35.1 ± 0.7 ^A^	36.7 ± 8.2 ^Aa^	30.3 ± 6.1 ^Aa^	32.3 ± 6.6 ^Aa^	29.8 ± 3.4 ^Aa^
	9	N/A	33.8 ± 3.4 ^a^	30.7 ± 0.5 ^ab^	37.9 ± 4.2 ^a^	34.6 ± 2.2 ^b^
	21	N/A	36.0 ± 1.2 ^a^	33.2 ± 2.4 ^b^	32.7 ± 2.5 ^a^	30.7 ± 4.5 ^b^
Fe	0	54.5 ± 4.4 ^A^	61.8 ± 5.4 ^Aa^	52.8 ± 4.0 ^Aa^	59.1 ± 5.4 ^ABa^	57.7 ± 4.4 ^ABa^
	9	N/A	61.6 ± 1.7 ^a^	62.3 ± 5.0 ^a^	62.6 ± 0.8 ^a^	60.3 ± 4.5 ^a^
	21	N/A	60.6 ± 3.0 ^a^	62.4 ± 10 ^a^	55.8 ± 5.1 ^a^	59.5 ± 0.9 ^a^
Cu	0	163.7 ± 7.8 ^A^	170.0 ± 5.5 ^Aa^	173.6 ± 4.5 ^Aa^	158.8 ± 4.7 ^Aa^	167.4 ± 8.6 ^Aa^
	9	N/A	167.5 ± 1.9 ^a^	166.8 ± 5.2 ^a^	170.8 ± 0.3 ^b^	150.6 ± 8.7 ^a^
	21	N/A	178.1 ± 1.7 ^a^	173.7 ± 1.0 ^a^	173.2 ± 1.2 ^b^	170.9 ± 3.0 ^a^
Zn	0	58.3 ± 4.6 ^A^	57.9 ± 5.5 ^Aa^	52.8 ± 4.0 ^Aa^	55.6 ± 5.3 ^Aa^	60.9 ± 4.3 ^Aa^
	9	N/A	54.7 ± 4.4 ^a^	61.4 ± 6.3 ^a^	57.0 ± 7.1 ^a^	65.9 ± 7.1 ^a^
	21	N/A	60.5 ± 8.3 ^a^	60.5 ± 7.7 ^a^	55.8 ± 7.8 ^a^	63.3 ± 6.2 ^a^
Se	0	11.7 ± 0.2 ^A^	13.5 ± 2.7 ^Aa^	11.3 ± 0.3 ^Aa^	11.5 ± 0.5 ^Aa^	14.0 ± 1.7 ^Aa^
	9	N/A	13.9 ± 2.6 ^a^	13.4 ± 2.5 ^a^	15.2 ± 0.4 ^b^	11.3 ± 0.1 ^a^
	21	N/A	14.2 ± 0.8 a	11.7 ± 0.1 a	11.2 ± 0.6 a	11.5 ± 0.3 a

^1^ Data are expressed as mean ± standard deviation (*n* = 3). Different superscript capital letters indicate statistically significant differences (*p* ≤ 0.05) between treatments on day 0. Different superscript lowercase letters indicate statistically significant differences (*p* ≤ 0.05) between conservation days within the same treatment and mineral.

## Data Availability

Not applicable.

## Supplementary Materials

The following supporting information can be downloaded at: https://www.mdpi.com/article/10.3390/molecules28124675/s1, Table S1: Free fatty acid content (mg/100 mL) of Brazil nut beverages treated with HPH and pasteurization under cold storage (5 °C); Table S2: HPLC retention times, UV/Vis spectra, and MS spectral data of individual phenolic compounds in the Brazil nut beverage; Table S3: Antioxidant capacity of Brazil nut beverage treated with HPH and pasteurization under cold storage for 21 days.
