# Supplementary material for: Brazil Nut (Bertholletia excelsa) Beverage Processed by High-Pressure Homogenization: Changes in Main Components and Antioxidant Capacity during Cold Storage
Source: Molecules. 2023 Jun 9;28(12):4675. doi: 10.3390/molecules28124675 (PMC10305451; doi:10.3390/molecules28124675)
Supplement: Supplementary file 1 [file molecules-28-04675-s001.zip › molecules-2429598-supplementary.pdf]

Supplementary Material

Table S1. Free fatty acid content[1] (mg/ 100 mL) of Brazil nut beverages treated by HPH and pasteurization under cold storage (5 °C)

Day	BNB	PAS	T1	T2	T3
0	7.01 ±0.31^{AB}	8.47 ±0.6^{Bab}	7.88 ±0.33^{ABa}	6.87 ±0.41^{Aa}	7.23 ±0.26^{ABa}
9		10.1 ±0.6^{b}	8.98 ±0.58^{a}	9.37 ±0.21^{b}	9.08 ±0.56^{b}
21		6.85 ±0.21^{a}	6.99 ±0.5^{a}	6.75 ±0.14^{a}	7.47 ±0.13^{a}

[1]Data are expressed as mean ± standard deviation (n = 2). Different superscript capital letters indicate statistically significant differences ($p \leq 0.05$) between treatments in day 0. Different superscript lower-case letters indicate statistically significant differences ($p \leq 0.05$) between conservation days within the same treatment.

Table S2. HPLC retention times, UV/Vis λ_{max} and MS spectral data of individual phenolic compounds in the Brazil nut beverage.

Peak	Phenolic compound	RT (min)	UV/Vis λ_{max}	[M-H]- m/z	MS/MS
1	Gallic acid	5.7	274	169.01	125.02, 107.01, 97.03, 79.02, 69.03, 51.02, 41.04
2	Gallic acid derivative	6.2	272	187	125.02, 169.01
3	Catechin	20.9	230, 280	289.1	136.8, 150.7, 160.8
4	Catechin derivative	22.2	282	-	289.1
5	4-hydroxybenzoic acid	26.9	252	137.03	106.64, 93.03
6	Vanillic acid	29.0	259, 292	167.03	152.01, 108.02
7	Epicatechin	29.9	279	289.1	109.01, 121.01, 123.03, 125.01, 137.00
8	Vanillin	31.6	274, 309	151.05	137.05, 123.05, 109.0, 81.0
9	Catechin gallate	32.9	231.8, 280.1, (324.8)	441.03	109.01, 125.00, 168.98, 289.03
10	p-Coumaric acid	35.0	227, 310	164.05	119.05, 91.05
11	Ferulic acid	36.0	(292), 323	193.1	177.1, 161, 133.1
12	Ellagic acid derivative	37.6	252, 360	447	301, 257, 229
13	Quercetin	38.6	232, 323	301	179, 151

Table S3. Antioxidant capacity[1] of Brazil nut beverage treated by HPH and pasteurization under cold storage during 21 days.

**Sample**	**Cold storage (day)**					
	**0**	**2**	**5**	**9**	**15**	**21**
DPPH						
BNB	8.95 ±0.6^{B}					
PAS	6.36 ±0.2^{Aa}	7.47 ±0.1^{bc}	7.12 ±0.1^{b}	7.67 ±0.2^{c}	6.54 ±0.2^{a}	7.60 ±0.1^{c}
T1	6.56 ±0.1^{Aa}	6.70 ±0.2^{ab}	7.42 ±0.1^{c}	6.97 ±0.1^{b}	7.41 ±0.1^{c}	6.43 ±0.0^{a}
T2	6.55 ±0.1^{Aab}	6.79 ±0.3^{bc}	7.98 ±0.1^{e}	6.99 ±0.1^{cd}	6.35 ±0.1^{a}	7.34 ±0.0^{d}
T3	6.18 ±0.3^{Aa}	7.62 ±0.1^{c}	7.17 ±0.1^{bc}	7.56 ±0.1^{bc}	6.84 ±0.1^{abc}	6.73 ±0.7^{abc}
ABTS						
BNB	4.56 ±0.1^{B}					
PAS	2.87 ±0.0^{Aa}	3.11 ±0.0^{c}	3.06 ±0.0^{bc}	3.27 ±0.0^{d}	3.03 ±0.0^{b}	3.23 ±0.0^{d}
T1	2.86 ±0.0^{Aa}	2.99 ±0.0^{ab}	3.15 ±0.0^{bc}	3.28 ±0.1^{c}	3.07 ±0.1^{b}	2.87 ±0.1^{a}
T2	2.84 ±0.0^{Aa}	2.93 ±0.0^{ab}	3.18 ±0.1^{c}	3.07 ±0.1^{bc}	2.78 ±0.1^{a}	2.88 ±0.0^{a}
T3	2.76 ±0.3^{Aa}	3.13 ±0.0^{bc}	3.08 ±0.0^{abc}	3.37 ±0.1^{c}	2.89 ±0.0^{ab}	3.00 ±0.0^{ab}

Data are expressed as mean ± standard deviation (n = 3). Different superscript capital letters indicate statistically significant differences ($p \leq 0.05$) between treatments in day 0. Different superscript lower-case letters indicate statistically significant differences ($p \leq 0.05$) between conservation days within the same treatment.
